# A risk prediction model for poor joint function recovery after ankle fracture surgery based on interpretable machine learning

**DOI:** 10.3389/fmed.2025.1553274

**Published:** 2025-06-26

**Authors:** Congyang Li, Chenggang Wang, Jiru Zhang, Wenjun Zheng, Jing Shi, Li Li, Xuezhi Shi

**Affiliations:** ^1^Department of Orthopaedics, Lu’an Hospital of Anhui Medical University, Lu’an, China; ^2^Wound Stoma Care Clinic, Lu’an Hospital of Anhui Medical University, Anhui, China; ^3^Department of Science and Education, Lu’an Hospital of Anhui Medical University, Lu’an, China; ^4^Nursing Department, Lu’an Hospital of Anhui Medical University, Lu’an, China

**Keywords:** ankle fracture, poor functional recovery, machine learning, interpretability analysis, prediction

## Abstract

**Objective:**

Currently, there is no individualized prediction model for joint function recovery after ankle fracture surgery. This study aims to develop a prediction model for poor recovery following ankle fracture surgery using various machine learning algorithms to facilitate early identification of high-risk patients.

**Methods:**

A total of 750 patients who underwent ankle fracture surgery at Lu’an Hospital Affiliated to Anhui Medical University between January 2018 and December 2023 were followed up. The collected data were chronologically divided into a training set (599 cases) and a test set (151 cases). Feature variables were selected using the Boruta algorithm, and five machine learning algorithms (logistic regression, random forest, extreme gradient boosting, support vector machine, and lasso-stacking) were employed to construct models. The performance of these models was compared on both the training and test sets to select the best-performing model. The decision basis of the optimal model was further analyzed using Shapley Additive Explanation (SHAP) and Local Interpretable Model-Agnostic Explanations (LIME).

**Results:**

In total, 12 characteristic variables were identified using the Boruta algorithm. Among the five machine learning models, random forest model: AUC (training set: 0.840, test set: 0.779), accuracy (training set: 0.781, test set: 0.742); SVM: AUC (training set: 0.809, test set: 0.768), accuracy (training set: 0.751, test set: 0.728); XGBoost: AUC (training set: 0.734, test set: 0.748), accuracy (training set: 0.668, test set: 0.722); logistic regression: AUC (training set: 0.672, test set: 0.691), accuracy (training set: 0.651, test set: 0.656); lasso-stacking model: AUC (training set: 0.877, test set: 0.791), accuracy (training set: 0.796, test set: 0.762). The PR curve and decision curve of the lasso-stacking model were better than those of other models. The lasso-stacking model had the best performance. SHAP analysis showed that functional exercise compliance, combined ligament injury, and open fracture accounted for the largest proportion of SHAP values and were the most important influencing factors.

**Conclusion:**

Through evaluation and comparison of the developed models, the lasso-stacking model demonstrated the best performance and is more suitable for predicting joint function recovery after ankle surgery. This model can be further validated externally and applied in clinical practice.

## 1 Introduction

Ankle fractures, often resulting from external violent factors such as falls, sports injuries, and traffic accidents, are prevalent among middle-aged populations (1–3) and currently rank as the third most common fracture type in orthopedic practice (4, 5). Recent studies have revealed a trend of increasing incidence rates (6–8), accompanied by high treatment costs. In the United States, the average cost of outpatient treatment for ankle fractures is $9,821, while the average cost of inpatient treatment exceeds $62,000 (9, 10), posing a significant economic burden on patients. Surgery remains the primary treatment modality for ankle fractures (9, 11). However, due to various factors such as patient-specific differences, fracture severity, surgical complications, and postoperative rehabilitation, patients with ankle fractures face risks of joint weakness, stiffness, and residual pain, which can negatively impact joint function recovery, postoperative quality of life, increase the risk of reoperation, and even lead to disability (12–16). Currently, most clinicians focus on surgical technique improvements, postoperative complication prevention, and early postoperative rehabilitation (17–19), while epidemiological aspects of postoperative joint recovery and factors influencing individualized recovery receive less attention. Understanding these influencing factors, early identification of high-risk populations, and timely intervention are crucial for ensuring normal postoperative joint function recovery in patients with ankle fractures. However, there is currently no research on the individualized prediction of postoperative joint function recovery in patients with ankle fractures.

As a pivotal branch of artificial intelligence, machine learning (ML) has gained increasing traction in medical research due to its strengths in non-linear modeling and high-dimensional data processing, particularly in surgical outcome prediction (20, 21). Unlike conventional statistical methods—such as logistic regression and Cox proportional hazards models, which rely on linear assumptions and manual variable selection—ML algorithms autonomously identify latent non-linear relationships and interaction effects within complex datasets, thereby enhancing predictive accuracy and robustness (22). These advantages have translated into notable successes in orthopedic surgical outcome prediction. For instance, Lex et al. (23) conducted a systematic review of predictive models for hip fracture outcomes, demonstrating that ML algorithms achieved superior discriminative performance for 1-year postoperative mortality prediction (mean area under the receiver operating characteristic curve [AUC]: 0.84) compared to traditional models (AUC: 0.79). Similarly, Cai et al. developed a stacked ensemble-based ML classifier to predict the Japanese Orthopedic Association recovery rate for patients with degenerative cervical myelopathy, reporting exceptional performance (AUC: 0.92, accuracy: 90.2%, sensitivity: 90.1%) that significantly outperformed conventional approaches (AUC: 0.78, accuracy: 79.3%, sensitivity: 65.0%) (24). Despite these advancements, the application of ML in predicting outcomes following ankle fracture surgery remains scarcely investigated. Therefore, in this study, we employ several classic machine learning algorithms to construct prediction models for poor postoperative recovery after ankle fractures. By comparing the performance of these models, we select the optimal one and conduct an interpretability analysis to determine the significant influencing factors.

This study aims to provide clinical guidance for early identification of high-risk populations, timely implementation of preventive measures, facilitation of rapid postoperative recovery, reduction of hospitalization costs, and optimization of healthcare resource allocation for ankle fracture patients. By bridging this research gap, we seek to empower clinicians with data-driven tools to improve patient outcomes and quality of life.

## 2 Methods

### 2.1 Study designs

This single-center, prospective observational study was conducted with the approval of the Ethics Committee of Lu′an Hospital of Anhui Medical University (2024LLKS-KY-044). All participants provided written informed consent. Data were collected in January 2018 using a structured questionnaire and a clinical electronic inpatient record system. Participants completed the questionnaire online through Questionnaire Star, and hematological indicators were obtained through the clinical electronic inpatient medical record system. The study was conducted in accordance with the ethical standards of the local IRB and the 1975 Declaration of Helsinki.

### 2.2 Patients and data collection

#### 2.2.1 Study population

The study population consisted of 750 patients who underwent ankle surgery at the orthopedic department of a tertiary hospital in Lu′an City, Anhui Province, between January 2018 and December 2023.

The inclusion criteria were as follows: (1) age ≥ 18 years; (2) radiographic evidence of ankle fracture requiring surgery; (3) no previous ankle surgery; (4) voluntary participation in the study with signed informed consent.

The exclusion criteria were as follows: (1) patients choosing conservative treatment; (2) missing data; (3) presence of functional or organic mental disorders with language communication barriers.

#### 2.2.2 Data collection

Based on literature review and expert consultation, researchers identified 31 potential factors influencing poor prognosis after ankle fracture surgery, including 23 categorical and 8 continuous variables. The operational definitions of the candidate predictors are as follows:

1. Age (years): Refers to patients over the age of 18 undergoing ankle fracture surgery. This is a continuous variable.
2. Gender: Male or female. This is a categorical variable.
3. Education level: Describes the educational background of patients undergoing ankle fracture surgery, including primary school or below, junior high to high school, and university or above. This is a categorical variable.
4. Varicose veins: Indicates whether patients undergoing ankle fracture surgery have concomitant varicose veins. This is a categorical variable.
5. Heart disease: Denotes whether patients undergoing ankle fracture surgery have comorbid heart disease. This is a categorical variable.
6. Cerebrovascular disease: Represents whether patients undergoing ankle fracture surgery have comorbid cerebrovascular disease. This is a categorical variable.
7. Diabetes: Signifies whether patients undergoing ankle fracture surgery have comorbid diabetes. This is a categorical variable.
8. Hypertension: Indicates whether patients undergoing ankle fracture surgery have comorbid hypertension. This is a categorical variable.
9. Smoking: Refers to whether there is a history of smoking recorded in the electronic nursing records of patients undergoing ankle fracture surgery. This is a categorical variable.
10. Alcohol consumption: Pertains to whether there is a history of alcohol consumption recorded in the electronic nursing records of patients undergoing ankle fracture surgery. This is a categorical variable.
11. Injury mechanism: Describes how the ankle fracture occurred, including car accidents, falls, and other mechanisms. This is a categorical variable.
12. Injury site: Denotes the surgical site of patients undergoing ankle fracture surgery, including left, right, or bilateral. This is a categorical variable.
13. Body mass index (kg/m2): Represents the body mass index of patients at the time of admission for ankle fracture surgery. This is a continuous variable.
14. Combined ligament injury: Indicates whether patients with ankle fractures have concomitant ligament injuries. This is a categorical variable.
15. Nerve injury: Signifies whether patients with ankle fractures have concomitant nerve injuries. This is a categorical variable.
16. Combined joint dislocation: Represents whether patients with ankle fractures have concomitant joint dislocation. This is a categorical variable.
17. Open fracture: Denotes whether the ankle fracture is an open fracture. This is a categorical variable.
18. Fracture type: Describes the severity of the ankle fracture, including single ankle fracture, double ankle fracture, and triple ankle fracture. This is a categorical variable.
19. Surgical waiting time (days): Represents the time from injury to surgery for patients with ankle fractures. This is a continuous variable.
20. Perioperative use of blood-activating and stasis-removing drugs: Indicates whether patients with ankle fractures take blood-activating and stasis-removing drugs during the perioperative period. This is a categorical variable.
21. Postoperative hemoglobin (g/L): Signifies the hemoglobin value from the first hematological examination after ankle fracture surgery. This is a continuous variable.
22. Postoperative albumin (g/L): Represents the albumin value from the first hematological examination after ankle fracture surgery. This is a continuous variable.
23. Postoperative red blood cell count (×10^12^/L): Denotes the red blood cell count from the first hematological examination after ankle fracture surgery. This is a continuous variable.
24. Postoperative drainage tube: Indicates whether patients undergoing ankle fracture surgery have a postoperative incision drainage tube. This is a categorical variable.
25. Operation time (minutes): Represents the difference between the start and end times recorded in the electronic anesthesia record for ankle fracture surgery. This is a continuous variable.
26. Length of hospital stay (days): Describes the duration of the hospital stay for patients undergoing ankle fracture surgery. This is a continuous variable.
27. Venous thrombosis: Indicates whether venous thrombosis occurs during hospitalization for ankle fracture patients, as detected by ultrasound examination (25, 26). This is a categorical variable.
28. Surgical site infection: Represents whether a surgical site infection occurs after ankle fracture surgery. Surgical site infection is diagnosed based on the Guidelines for the Prevention of Surgical Site Infection from the Centers for Disease Control and Prevention and the Healthcare Infection Control Practices Advisory Committee (27, 28). This is a categorical variable.
29. The American Society of Anesthesiologists (ASA) Classification: Describes the ASA classification recorded in the electronic anesthesia record for ankle fracture surgery. ASA assesses patients’ physical status and surgical risk before anesthesia, ranging from Class 1 to Class 6, for evaluating surgical risk (29, 30). This is a continuous variable.
30. Postoperative pain level after functional exercise: Represents the average pain score recorded using The Numeric Rating Scale (NRS) in the electronic nursing records after each rehabilitation exercise guided by a therapist following ankle fracture surgery. Scores 1–3 indicate mild pain, 4–6 moderate pain, and 7–9 severe pain. This is a continuous variable.
31. Postoperative functional exercise compliance (PFEC): This refers to the compliance of patients with ankle fractures in performing rehabilitation exercises after being instructed by a rehabilitation therapist following surgery. Assessment is conducted on the day of the patient’s discharge using the Orthopedic Rehabilitation Exercise Compliance Scale. Developed by Chinese scholars Tan et al., the scale consists of three dimensions: compliance related to psychological persistence in exercise, compliance related to active learning and persistence in exercise, and compliance related to physical persistence in exercise. It includes 15 items rated on a 1–5 Likert scale, with a total score of 75 points; a higher score indicates better patient compliance with functional exercises. Scores ≤20 indicate low compliance, scores between 20 and 55 points indicate partial compliance, and scores ≥50 indicate high compliance. The total Cronbach’s α coefficient for this scale is 0.930, with coefficients of 0.920, 0.842, and 0.851 for each respective dimension (31). This is a continuous variable.

#### 2.2.3 Criteria for diagnosing poor recovery

The American Orthopaedic Foot and Ankle Society Ankle-Hindfoot Scale (AOFAS) was used to evaluate the functional recovery of patients’ ankles 3 months after surgery. This scale, proposed by KITAOKA et al. in 1994 (32), has been widely applied in many countries (33, 34). It is primarily designed to assess ankle function. The scale ranges from 0 to 100 points, with excellent (90–100 points), good (75–89 points), moderate (50–74 points), and poor (<50 points) categories. A higher score indicates better ankle function. In this study, patients with an AOFAS score below 75 at 3 months post-ankle surgery were diagnosed as having a poor prognosis (poor prognosis label = 1, non-poor prognosis = 0).

### 2.3 Feature selection and model construction

**Figure 1:**
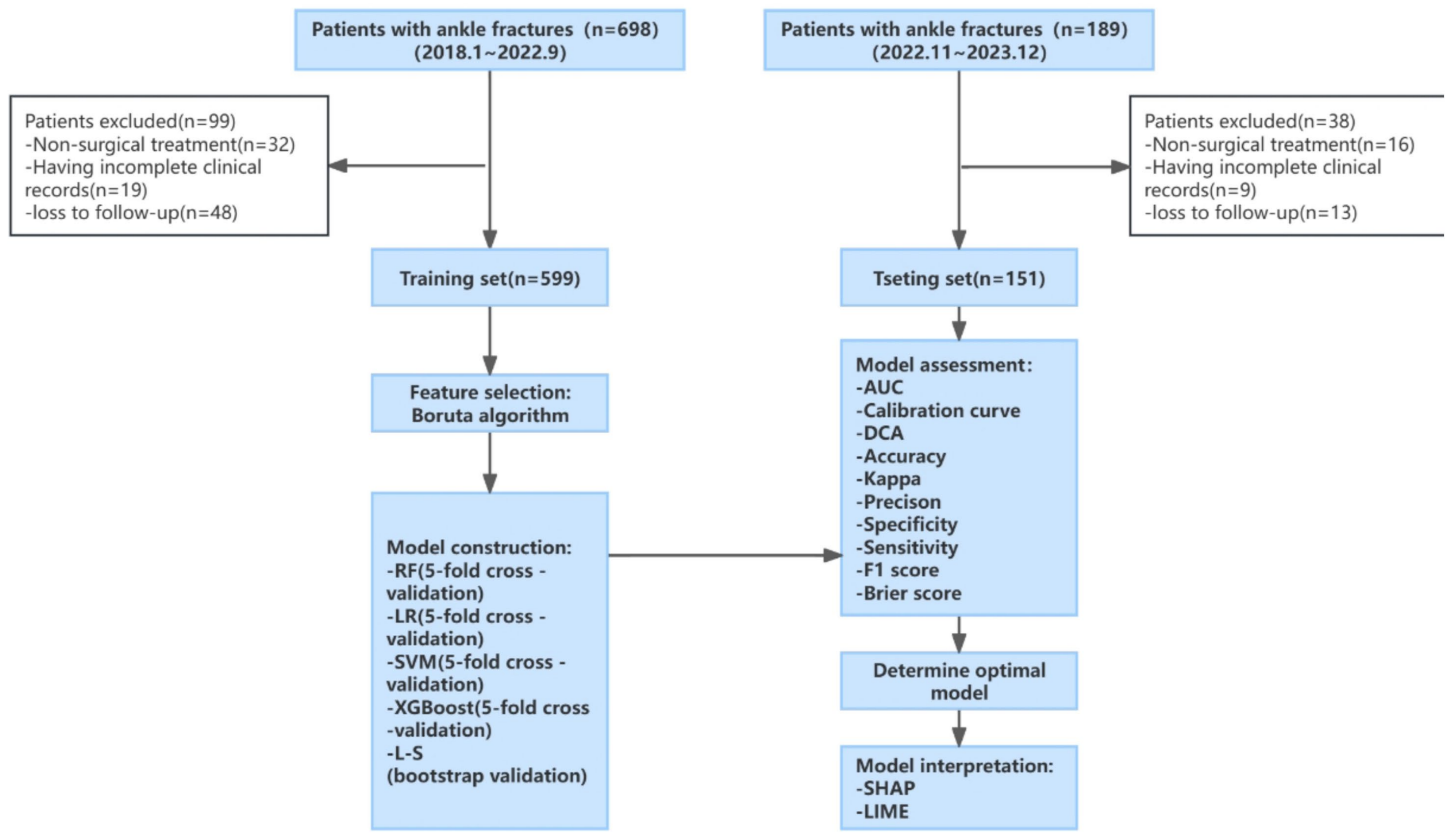
Study flow chart. LR: RF, random forest; logistic regression; SVM, support vector machine; XGBoost, eXtreme gradient boosting; L-S, Lasso-Stacking; AUC, area under the receiver operating characteristic curve; DCA, decision curve analysis; SHAP, Shapley additive explanations; LIME, Local Interpretable Model-Agnostic Explanations.

The flowchart of this study is shown in Figure 1. The included patients were divided into two groups chronologically (80% training set, 20% test set). The training set data was used to build the model, while the test set was used to evaluate the performance of the model.

In this study, the Boruta algorithm was employed for feature variable selection. The core steps of this method are divided into constructing shadow features and random forest voting. Shadow features are copies of the original features, where the values are randomly rearranged to eliminate their correlation with the outcome variable. The Boruta algorithm combines shadow features and original features to construct a new dataset, utilizing a random forest model to determine the importance of each feature in the new dataset (35). Random permutation of attribute values among objects leads to a decrease in classification accuracy, and the Z-score, which is the average accuracy loss divided by its standard deviation, serves as an indicator to measure feature importance for variable selection.

In this study, five machine learning algorithms were employed to construct prediction models, namely: random forest (RF), support vector machine (SVM), eXtreme gradient boosting (XGBoost), logistic regression (LR), and least absolute shrinkage and selection operator stacking (lasso-stacking). Among these, lasso-stacking is a stacking ensemble model based on the first four models, with lasso serving as the meta-model. The first four models underwent five-fold cross-validation, while the lasso-stacking model adopted Bootstrap resampling for cross-validation to ensure the stability and accuracy of the models.

The hyperparameter optimization methods for the random forest, SVM, XGBoost, and lasso-stacking algorithms were implemented as follows:

1. Random forest: Within the tidymodels framework, hyperparameter tuning was performed using the randomForest engine through a 5-fold cross-validation grid search. The key hyperparameters optimized included: mtry (number of features considered at each split): Initially set as sqrt(p), with optimal values selected between 2 and 10; min_n (minimum node size): Optimized within the range of 20 to 50; trees (number of trees in the forest): Evaluated across 200–500 candidate values. The grid search for all hyperparameters was executed using the tidymodels framework, with AUC (area under the ROC curve) as the evaluation metric. The optimal parameter combination was identified as mtry = 33, min_n = 39, and trees = 235, achieving an ROC_AUC of 0.692.
2. XGBoost: Hyperparameter optimization was performed using the xgboost engine within the tidymodels framework, employing a 5-fold cross-validated grid search. This approach enabled systematic evaluation of model performance across diverse hyperparameter combinations to identify optimal settings. The following hyperparameters were prioritized for tuning: mtry (number of features evaluated at each node split), trees (total number of decision trees in the ensemble), min_n (minimum sample size required for terminal leaf nodes), tree_depth (maximum permissible depth of individual trees), learn_rate (learning rate governing stepwise contributions of trees to final predictions), loss_reduction (minimum loss reduction required for node splitting), sample_size (proportion of training samples utilized per iteration), and stop_iter (number of iterations without improvement for early stopping). All grid search procedures were executed under the tidymodels framework, with AUC (area under the ROC curve) serving as the primary evaluation metric. With trees fixed at 1,000 and stop_iter set to 25, the optimal parameter combination was identified as mtry = 3, min_n = 9, tree_depth = 2, learn_rate = 0.00564, loss_reduction = 0.0351, and sample_size = 0.871, achieving an ROC_AUC of 0.639.
3. SVM: The model was implemented using the svm_rbf() function from the tidymodels framework with the kernlab engine. A radial basis function (RBF) kernel was employed, with two critical hyperparameters—cost (C) and rbf_sigma (γ)—optimized via a 5-fold cross-validated grid search over predefined parameter spaces. The cost parameter governed the penalty for misclassifications, while rbf_sigma determined the width of the RBF kernel, thereby influencing model flexibility. Hyperparameter tuning was guided by maximization of the area under the ROC curve (AUC) to ensure an optimal balance between model complexity and generalization capability. The optimal parameter combination was identified as cost (C) = 5.15 and rbf_sigma (γ) = 0.00905.
4. Lasso-stacking: A stacked ensemble model was constructed within the tidymodels framework, incorporating random forest, support vector machine, XGBoost, and logistic regression as base learners, with a lasso-regularized logistic regression model serving as the meta-learner. Model validation was conducted using bootstrap resampling-based cross-validation, with ROC_AUC adopted as the performance metric. Hyperparameter tuning focused on the lasso penalty parameter (λ), which governs feature sparsity in the meta-learner. The final model selected λ = 0.03316, yielding a sparse ensemble where only a subset of base learners contributed to predictions. The stacked ensemble demonstrated a mean cross-validated ROC AUC of 0.703.

### 2.4 Model assessment

In this study, the performance of the models was evaluated separately on the training and test sets to determine the best model. Initially, the receiver operating characteristic (ROC) curve was plotted, and the area under the ROC curve (AUC) was calculated to quantify its discriminatory performance. The calibration of the model was assessed by plotting a calibration curve and computing the Brier score. Subsequently, the precision–recall (P–R) curve was utilized to further evaluate the model’s discriminatory ability by plotting the relationship between positive predictive value (PPV) and true positive rate (TPR) for all thresholds. Additionally, the clinical decision curve (DCA) was employed to assess the clinical net benefit of each model. The DeLong test was used to evaluate the robustness of the model. Finally, additional metrics such as accuracy, Kappa, precision, specificity, sensitivity, and F1 score were used to evaluate the predictive capabilities of the models (36).

### 2.5 Model interpretability analysis

In the optimal model, the contribution and significance of each feature variable to the outcome were determined based on the Shapley Additive Explanation (SHAP) values. Furthermore, Local Interpretable Model-Agnostic Explanations (LIME) were used to provide further interpretation of the model (37–40).

### 2.6 Quality control

To reduce bias in the data collection process, which may affect the research results, all objective data were obtained from electronic medical records and electronic nursing records and entered into the database through a double-check process. Unified training was conducted before the questionnaire survey, standardized sentences were used during the survey, and questionnaire scores were also entered into the database using a double cross-check. The database is maintained by a designated person, and once data are cross-checked and entered, they cannot be changed. In the process of model construction, to reduce the bias caused by time division, we tested the robustness of the model using random stratification of the data.

### 2.7 Statistical analysis

Descriptive and differential statistical analyses were conducted using SPSS 26.0 software, while model construction was performed using R 4.3.2 software. Measurement data conforming to a normal distribution were described using mean ± standard deviation, and comparisons between groups were made using the t-test. Count data were described using frequencies and rates, and comparisons between groups were performed using the chi-square test or Fisher’s exact probability method. The R package “Boruta” was used for Boruta analysis, while “tidymodels” and “stacks” were employed for model training. Stacking utilized bootstrap resampling for hyperparameter tuning, while grid search methods were used for hyperparameter tuning in the remaining models. The “fastshap,” “shapviz,” and “lime” packages were utilized to complete the interpretability analysis of SHAP and LIME. A *p*-value less than 0.05 was considered statistically significant.

## 3 Results

### 3.1 Baseline characteristics

In this study, a total of 750 patients undergoing ankle surgery were included, with a mean age of (49.93 ± 15.84) years. Among them, 414 were men and 336 were women. Poor postoperative recovery occurred in 248 patients, with an incidence rate of 33.1%. The data were divided into the training set and the testing set in chronological order at a ratio of 8:2. A comparison of differences between the two datasets revealed no significant differences among the variables, indicating comparability between the two datasets. Detailed baseline patient characteristics and the results of the difference comparison are presented in Table 1.

**Table 1:** Comparative analysis of data variability between the train set and the test set.

Clinical characteristics	Total (*n* = 750)	Training set (*n* = 599)	Testing set (*n* = 151)	*χ^2^/t/Fisher*	*p*
Age (years)	49.93 ± 15.84	49.78 ± 15.67	50.54 ± 16.54	−0.53	0.60
Gender (%)				2.34	0.13
Male	414 (55.2)	339 (56.6)	75 (49.7)		
Female	336 (44.8)	260 (43.4)	76 (50.3)		
Education Level (%)				4.45	0.11
Primary school and below	279 (37.2)	217 (36.2)	62 (41.1)		
Middle school to high school	356 (47.5)	282 (47.1)	74 (49.0)		
University and above	115 (15.3)	100 (16.7)	15 (9.9)		
VV (%)				0.65	0.42
Yes	78 (10.4)	65 (10.9)	13 (8.6)		
No	672(89.6)	534 (89.1)	138 (91.4)		
HD (%)				0.17	0.68
Yes	116 (15.5)	91 (15.2)	25 (16.6)		
No	634 (84.5)	508 (84.8)	126 (83.4)		
CD (%)				0.17	0.68
Yes	184 (24.5)	145 (24.2)	39 (25.8)		
No	566 (75.5)	454 (75.8)	112 (74.2)		
Diabetes (%)				0.73	0.39
Yes	174 (23.2)	135 (22.5)	39 (25.8)		
No	576 (76.8)	464 (77.5)	112 (74.2)		
Hypertension (%)				5.27	0.02
Yes	237 (31.6)	201 (33.6)	36 (23.8)		
No	513 (68.4)	398 (66.4)	115 (76.2)		
IM (%)				1.73	0.42
Fall	474 (63.2)	376 (62.8)	98 (64.9)		
Traffic accident injury	141 (18.8)	118 (19.7)	23 (15.2)		
Others	135(18.0)	105(17.5)	30(19.9)		
Smoking (%)				0.27	0.61
Yes	186 (24.8)	151 (25.2)	35 (23.2)		
No	564 (75.2)	448 (74.8)	116 (76.8)		
Alcohol consumption (%)				0.03	0.86
Yes	203 (27.1)	163 (27.2)	40 (26.5)		
No	547 (72.9)	436 (72.8)	111 (73.5)		
IS (%)				0.46	0.79
Left	386 (51.5)	312 (52.1)	74 (49.0)		
Right	335 (44.7)	264 (44.1)	71 (47.0)		
Both	29 (3.9)	23 (3.8)	6 (4.0)		
BMI (kg/m^2^)	24.18 ± 3.39	24.20 ± 3.38	24.11 ± 3.42	0.29	0.78
CLI (%)				1.03	0.31
Yes	275 (36.7)	225 (37.6)	50 (33.1)		
No	475 (63.3)	374 (62.4)	101 (66.9)		
FT (%)				3.10	0.21
Unilateral ankle fracture	597(79.6)	473 (79.0)	124 (82.1)		
Bilateral ankle fracture	38(5.1)	28 (4.7)	10 (6.6)		
Trimalleolar ankle fracture	115 (15.3)	98 (16.3)	17 (11.3)		
NI (%)				1.23	0.27
Yes	145 (19.3)	111 (18.5)	34 (22.5)		
No	605 (80.7)	488 (81.5)	117 (77.5)		
OF (%)				1.12	0.29
Yes	146 (19.5)	112 (18.7)	34 (22.5)		
No	604 (80.5)	487 (81.3)	117 (77.5)		
CJD (%)				0.26	0.61
Yes	140 (18.7)	114 (19.0)	26 (17.2)		
No	610 (81.3)	485 (81.0)	125 (82.8)		
SWT (days)	7.24 ± 3.40	7.26 ± 3.38	7.17 ± 3.50	0.30	0.76
PUBSD (%)				0.44	0.51
Yes	634 (84.5)	509 (85.0)	125 (82.8)		
No	116 (15.5)	90 (15.0)	26 (17.2)		
PHb (g/L)	118.46 ± 17.71	118.33 ± 17.40	119.01 ± 18.92	−0.42	0.76
PAlb (g/L)	41.39 ± 4.64	41.38 ± 4.63	41.41 ± 4.66	−0.07	0.95
PRBC (×10^12^/L)	4.03 ± 0.61	4.03 ± 0.60	4.03 ± 0.63	0.10	0.92
OT (min)	108.51 ± 45.31	108.99 ± 46.12	106.63 ± 42.03	0.57	0.57
PDT (%)				0.003	0.96
Yes	242 (32.2)	193 (32.2)	49 (32.4)		
No	508 (67.7)	406 (67.8)	102 (67.6)		
ASA (%)					0.41
1	671 (89.5)	538 (89.8)	133 (88.1)		
2	71 (9.5)	56 (9.3)	15 (9.9)		
3	8 (1.1)	5 (0.9)	3 (2.0)		
LHS (days)	15.10 ± 6.30	15.12 ± 6.27	15.06 ± 6.44	0.10	0.92
VT(%)				2.115	0.15
Yes	54 (7.2)	39 (6.5)	15 (9.9)		
No	696 (92.8)	560 (93.5)	136 (90.1)		
SSI (%)				1.63	0.20
Yes	35 (4.7)	25 (4.2)	10 (6.6)		
No	715 (95.3)	574 (95.8)	141 (93.4)		
PPLAFE (%)				0.63	0.74
Low	367 (48.9)	294 (49.1)	73 (48.3)		
Medium	325 (43.3)	261 (43.6)	64 (42.4)		
High	58 (7.7)	44 (7.3)	14 (9.3)		
PFEC (%)				1.58	0.46
Low	234 (31.2)	183 (20.6)	51 (33.8)		
Medium	327 (43.6)	268 (44.7)	59 (39.1)		
High	189 (25.2)	148 (24.7)	41 (27.1)		
PR (%)				<0.001	0.99
Yes	248 (33.1)	198 (33.1)	50 (33.1)		
No	502 (66.9)	401 (66.9)	101 (66.9)		

VV, Varicose Veins; HD, Heart Disease; CD, Cerebrovascular Disease; IM, Injury Mechanism; IS, Injury Site; BMI, Body Mass Index; CLI, Combined Ligament Injury; NI, Nerve Injury; CJD, Combined Joint Dislocation; OF, Open Fracture; FT, Fracture Type; SWT, Surgical Waiting Time; PUBSD, Perioperative Use of Blood-activating and Stasis-removing Drugs; PHb, Postoperative Hemoglobin; PAlb, Postoperative Albumin; PRBC, Postoperative Red Blood Cell Count; PDT, Postoperative Drainage Tube; OT, Operation Time; LHS, Length of Hospital Stay; VT, Venous Thrombosis; SSI, Surgical Site Infection; ASA, The American Society of Anesthesiologists; PPLAFE, Postoperative Pain Level After Functional Exercise; PFEC, Postoperative Functional Exercise Compliance.

### 3.2 Feature selection

**Figure 2:**
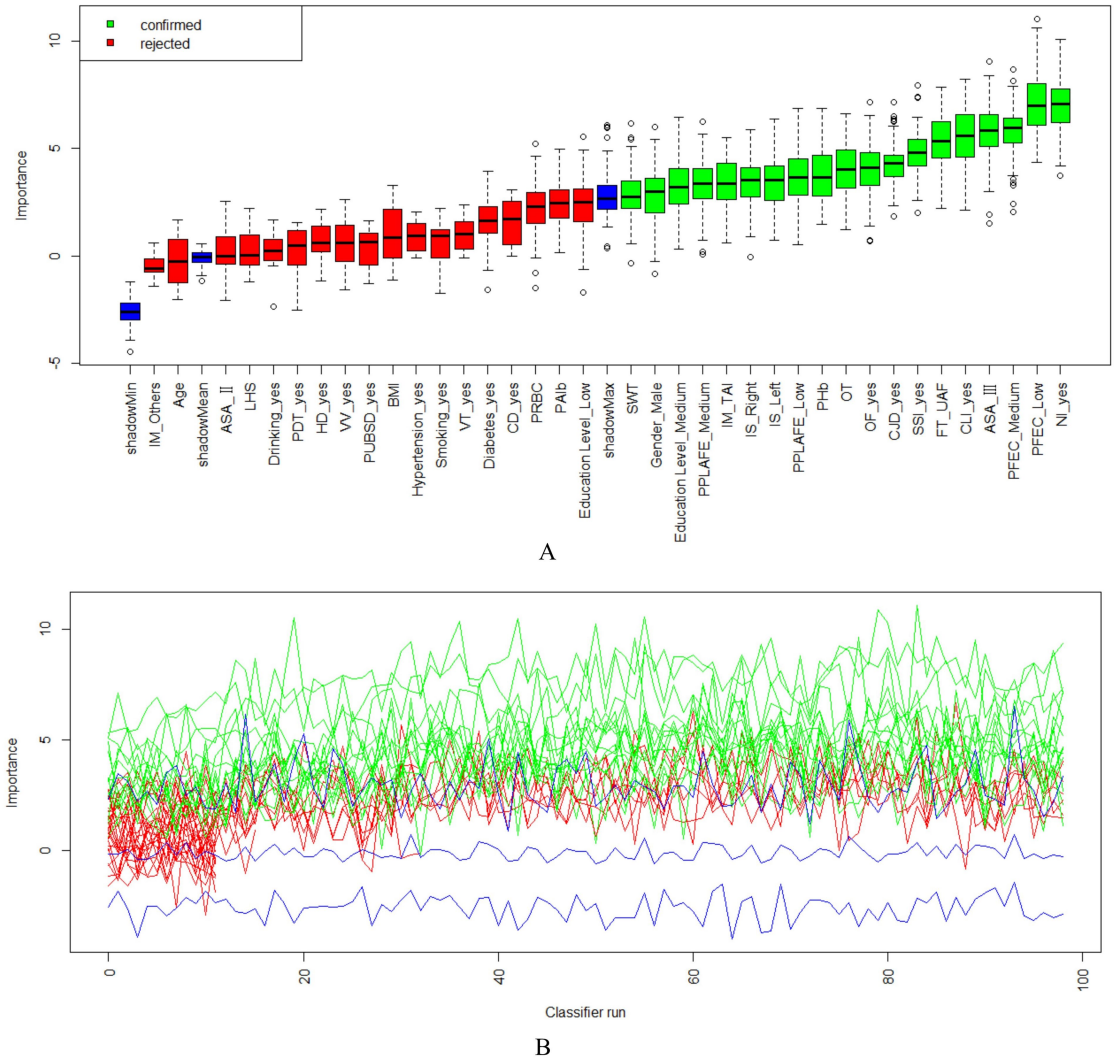
The Boruta algorithm feature screening graph comprises two sections; **(A)** the Boruta algorithm feature screening importance ranking of each variable and screening results; **(B)** the Z-score score change of each feature variable. IM, Injury Mechanism; LHS, Length of Hospital Stay; PDT, Postoperative Drainage Tube; HD, Heart Disease; VV, Varicose Veins; PUBSD, Perioperative Use of Blood-activating and Stasis-removing Drugs; VT, Venous Thrombosis; CD, Cerebrovascular Disease; PRBC, Postoperative Red Blood Cell Count; PAlb, Postoperative Albumin; Education Level_Low: Education Level_Primary school and below; Education Level_Medium: Education Level_Middle school to high school; Education Level_High: University and above; PPLAFE, Postoperative Pain Level After Functional Exercise; IS, Injury Site; BMI, Body Mass Index; CLI, Combined Ligament Injury; NI, Nerve Injury; CJD, Combined Joint Dislocation; OF, Open Fracture; FT, Fracture Type; SWT, Surgical Waiting Time; PHb, Postoperative Hemoglobin; OT, Operation Time; SSI, Surgical Site Infection; ASA, The American Society of Anesthesiologists; PFEC, Postoperative Functional Exercise Compliance.

As shown in Figure 2, using the Boruta algorithm, 12 potential predictors were selected based on the importance of their Z-scores. The selected predictors are as follows: postoperative albumin, operation time, injury mechanism, injury site, combined ligament injury, fracture severity, combined nerve injury, open fracture, combined joint dislocation, postoperative infection, pain level after postoperative rehabilitation exercise, and compliance with functional exercise.

### 3.3 Model construction, evaluation, and comparison

**Figure 3:**
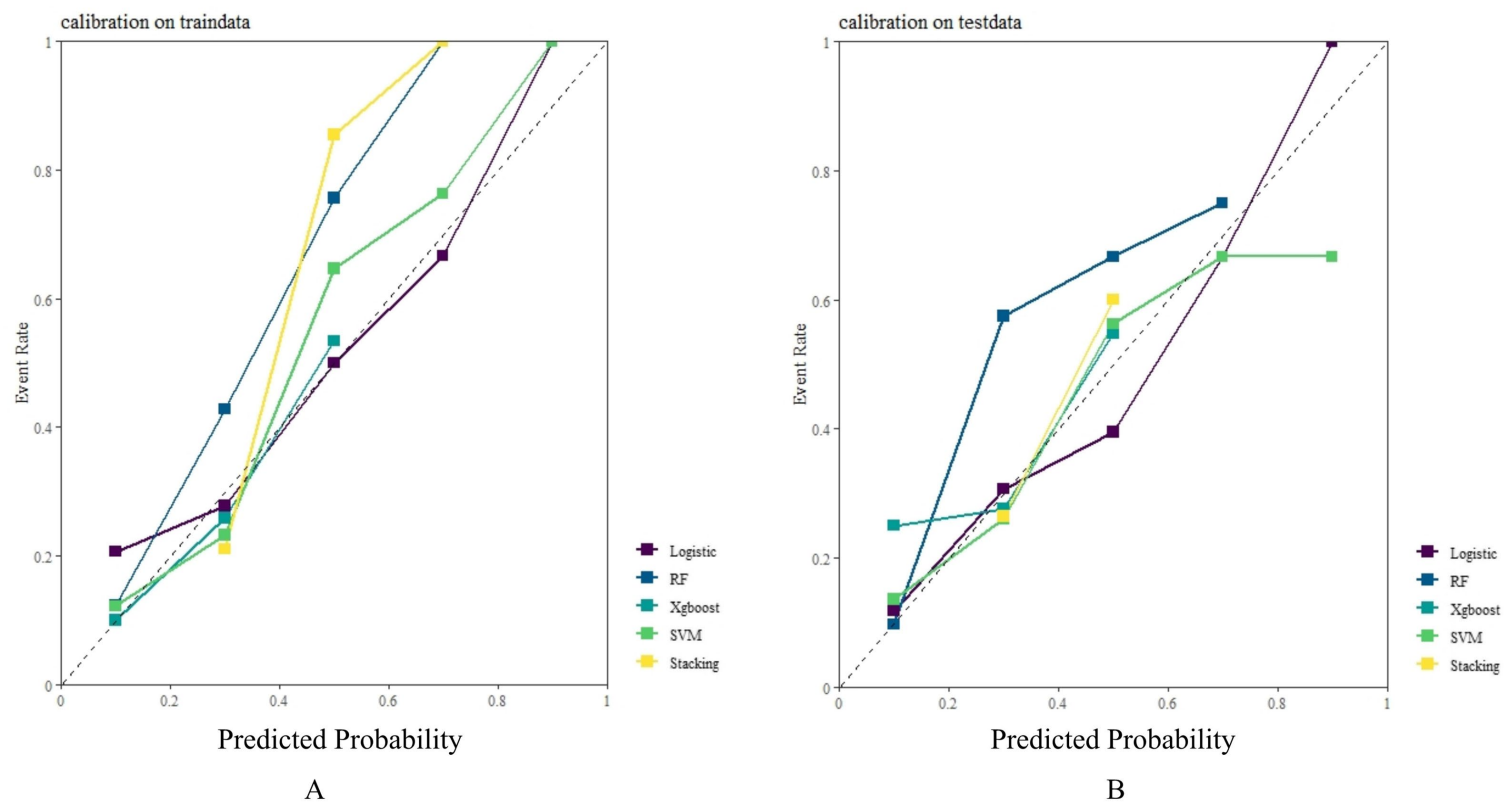
Model calibration curve: **(A)** Model calibration curves in the training set: **(B)** Model calibration curves in the testing set.

**Figure 4:**
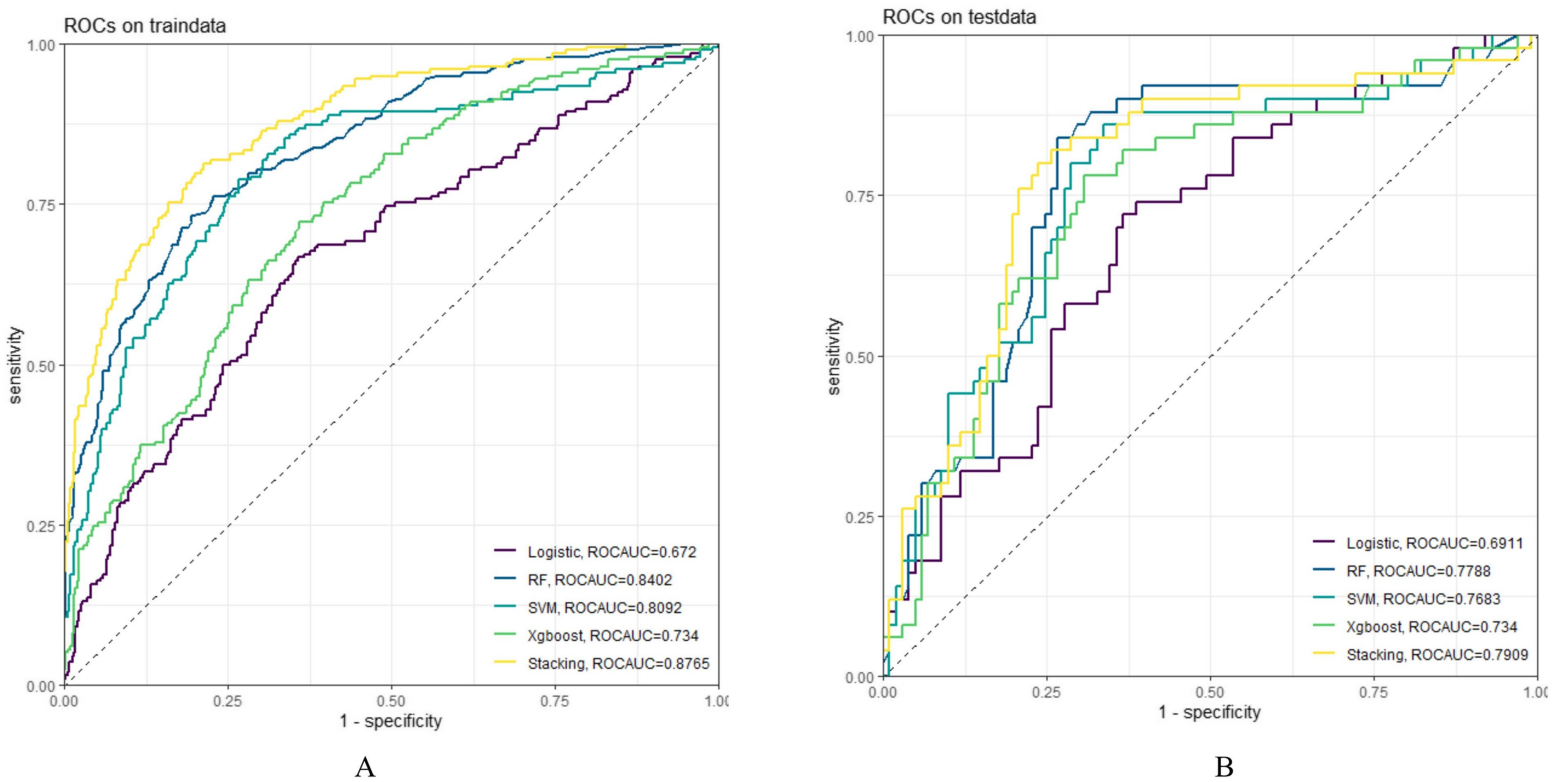
Model ROC curve; **(A)** ROC curve of the model in the training set; **(B)**: ROC curve of the model in the testing set; ROC, receiver operating characteristic.

**Figure 5:**
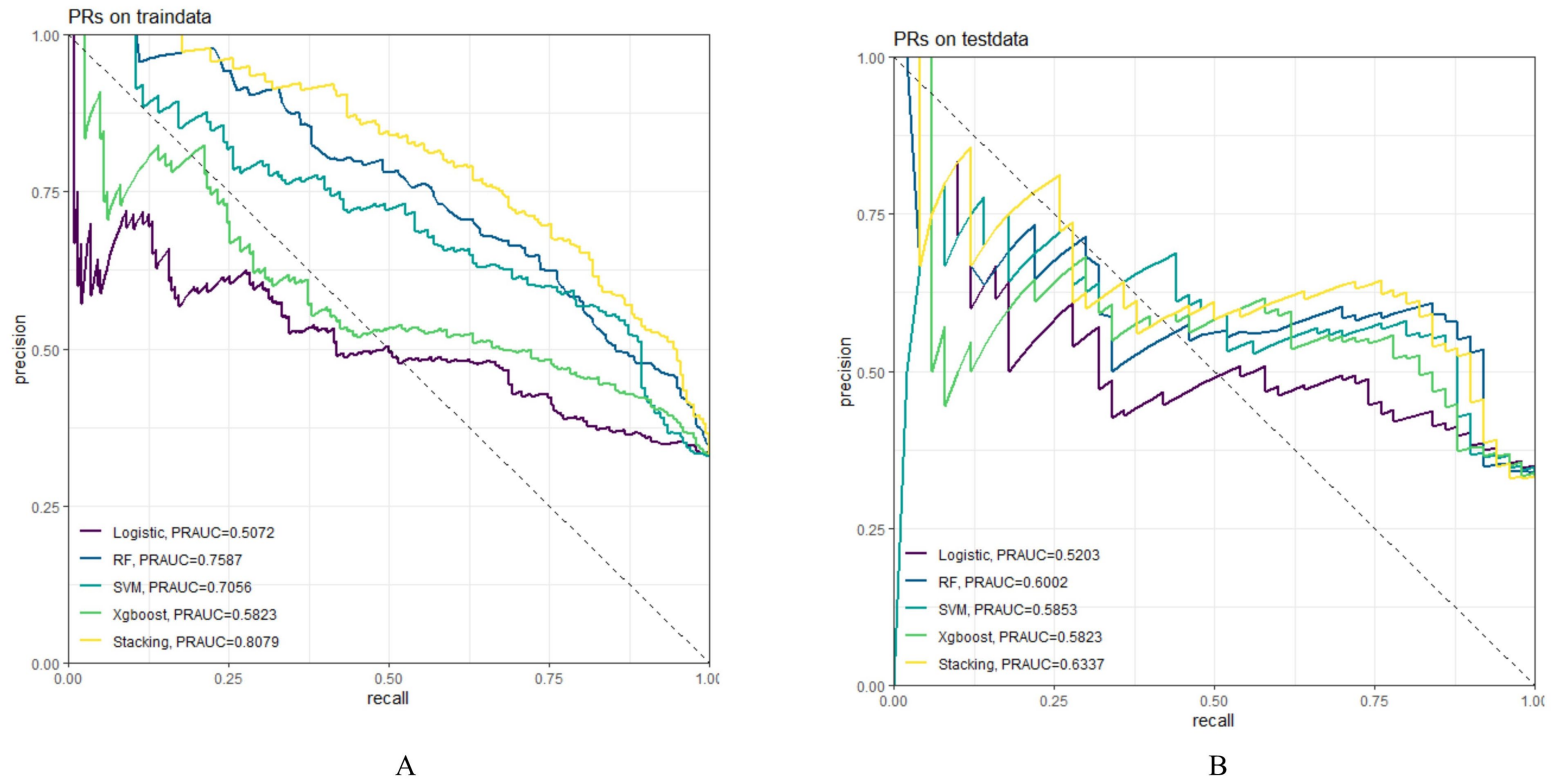
Model PR curve; **(A)** PR curve of the model in the training set; **(B)** PR curve of the model in the testing set; PR, precision-Recall.

**Figure 6:**
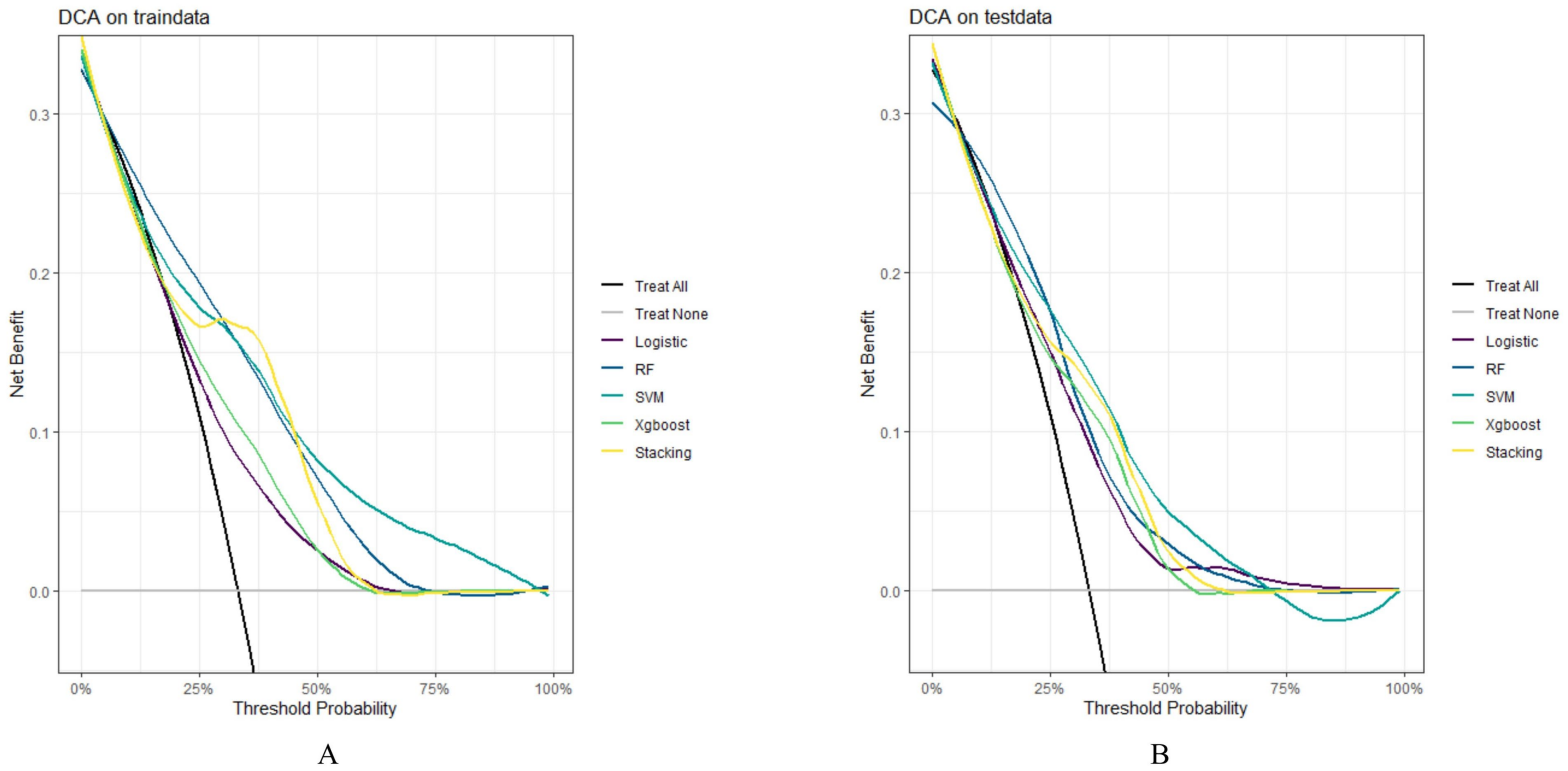
Model DCA curve; **(A)** DCA curves of the model in the training set; **(B)** DCA curves of the model in the testing set; DCA, decision curve analysis.

In both the training and testing sets, all models achieved accuracy and AUC values above 0.60, with Brier scores less than 0.25. The DeLong test *p*-values for AUC between the training and testing sets were all greater than 0.05 (Tables 2–4). Calibration curves indicated that all models demonstrated good calibration (Figure 3). The ROC curves for the training and testing sets are shown in Figure 4. Among the models, the L-S model exhibited the highest AUC (training set: 0.877, testing set: 0.791) and accuracy (training set: 0.796, testing set: 0.762), indicating strong and robust discriminatory ability. The LR model is often used as a traditional baseline model; therefore, the DeLong test was employed to compare the AUC of other models with both the L-S model and the LR model. In the training set, the Delong test *p*-values (both vs. the LR model and vs. the lasso-stacking model) were below 0.05. In the testing set, the DeLong test p-values compared to the LR model were less than 0.05 except for the SVM model, while p-values compared to the lasso-stacking model were greater than 0.05 except for the LR model. The Lasso-Stacking model also performed better or comparably to other models in terms of accuracy, Kappa, precision, specificity, sensitivity, and F1 score in both the training and testing sets. Additionally, the L-S model achieved the highest area under the PR curve among the five models (training set: 0.808, testing set: 0.634; Figure 5). Decision curve analysis revealed that the SVM model and the lasso-stacking model provided the highest clinical net benefit (Figure 6). In addition, the data were randomly grouped according to the ratio of 3:7 (dataset 1 and dataset 2), and the performance of the model in the two datasets was consistent with that observed in the training set and test set. Detailed results are presented in Supplementary Table S1S1 and S2 in the Appendix. DeLong test *p*-values > 0.05 indicate that each model demonstrated more robustness. Furthermore, the data were stratified based on demographic characteristics and fracture types. The performance of each model across different demographic characteristics is detailed in Supplementary Table S4 in the Appendix, and the performance of each model across different fracture types is detailed in Supplementary Table S5 in the Appendix. Accuracy and ROC curves of each model across different demographic characteristics and across different fracture types are compared in Appendix Supplementary Figures S1–S4. The comparison showed that the lasso-stacking model consistently exhibited the best performance. In conclusion, the lasso-stacking model emerged as the optimal model and appears most suitable for predicting poor recovery after ankle fracture surgery.

**Table 2:** Performance of five machine learning-based models for predicting poor joint recovery after ankle fracture in the training set.

Model	Delong test *p*-value (vs. LR model)	DeLong test *p*-value (vs. L-S model)	AUC(95%CI)	Accuracy	Kappa	Precision	Specificity	Sensitivity	F1 score	Brier score
RF	<0.001	<0.001	0.840(0.807 ~ 0.874)	0.781	0.521	0.650	0.805	0.732	0.689	0.169
LR	-	<0.001	0.672(0.626 ~ 0.719)	0.651	0.282	0.480	0.643	0.667	0.558	0.203
SVM	<0.001	<0.001	0.809(0.770 ~ 0.848)	0.751	0.481	0.593	0.733	0.788	0.677	0.169
XGBoost	<0.001	<0.001	0.734(0.692 ~ 0.776)	0.668	0.326	0.498	0.641	0.722	0.590	0.198
Lasso-stacking	<0.001	-	0.877(0.847 ~ 0.906)	0.796	0.566	0.654	0.788	0.813	0.725	0.18

AUC, area under the curve; 95%CI, confidence interval.

**Table 3:** Performance of five machine learning-based models for predicting poor joint recovery after ankle fracture in the testing set.

Model	DeLong test *p*-value (vs. LR model)	DeLong test *p*-value (vs. L-S model)	AUC(95%CI)	Accuracy	Kappa	Precision	Specificity	Sensitivity	F1 score	Brier score
RF	0.005	0.257	0.779(0.698 ~ 0.860)	0.742	0.448	0.590	0.752	0.720	0.649	0.190
LR	-	0.003	0.691(0.603 ~ 0.779)	0.656	0.307	0.486	0.624	0.720	0.581	0.199
SVM	0.053	0.314	0.768(0.686 ~ 0.851)	0.728	0.446	0.563	0.693	0.800	0.661	0.183
XGBoost	0.031	0.153	0.748(0.663 ~ 0.832)	0.722	0.430	0.557	0.693	0.780	0.650	0.198
Lasso-stacking	0.003	-	0.791(0.711 ~ 0.871)	0.762	0.502	0.606	0.743	0.800	0.690	0.193

AUC, area under the curve; 95%CI, confidence interval.

**Table 4:** Results of AUC DeLong test between training set and testing set of five machine learning.

Model	Training set		Testing set		*z*	*p*
	AUC	95%CI	AUC	95%CI		
RF	0.840	0.807 ~ 0.874	0.779	0.698 ~ 0.860	1.373	0.171
LR	0.672	0.626 ~ 0.719	0.691	0.603 ~ 0.779	−0.377	0.707
SVM	0.809	0.770 ~ 0.848	0.768	0.686 ~ 0.851	0.881	0.379
XGBoost	0.734	0.692 ~ 0.776	0.748	0.663 ~ 0.832	−0.287	0.775
Lasso-stacking	0.877	0.847 ~ 0.906	0.791	0.711 ~ 0.871	1.968	0.051

AUC, area under the curve; 95%CI, confidence interval.

### 3.4 Model interpretation

**Figure 7:**
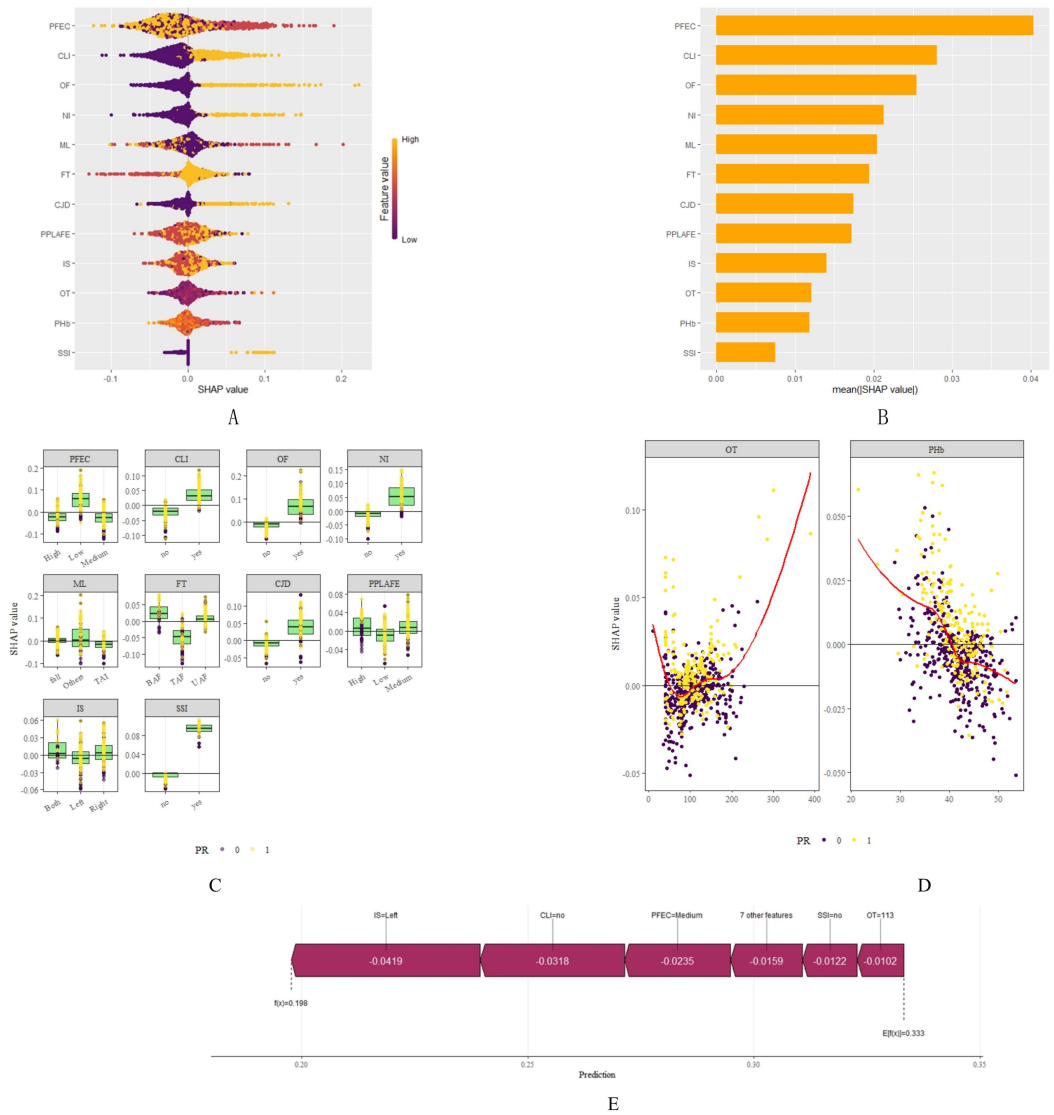
SHAP plots. **(A)** SHAP summary plot shows feature importance for each predictor of the Lasso-Stacking model in descending order. The upper predictors are more important to the model’s predictive outcome. For each patient’s Lasso-Stacking model, a point should be created for each feature attribute value. The distance of a point from the baseline SHAP value of zero indicates the strength of its effect on the model output. The points are coloured according to the value of the feature, with yellow representing high feature values and red representing low feature values. **(B)** Bar chart of mean absolute SHAP for each predictor of the Lasso-Stacking model in descending order **(C)** SHAP chart for each categorical variable. For each patient’s Lasso-Stacking model, a point should be created for each feature attribute value. The distance of a point from the baseline SHAP value of zero indicates the strength of its effect on the model output. Yellow for positive results, purple for negative results **(D)** SHAP chart for each continuous variable. For each patient’s Lasso-Stacking model, a point should be created for each feature attribute value. The distance of a point from the baseline SHAP value of zero indicates the strength of its effect on the model output. Yellow for positive results, purple for negative results **(E)** The force plots provide personalized feature attributions using one examples. IM, Injury Mechanism; IS, Injury Site; CLI, Combined Ligament Injury; NI, Nerve Injury; CJD, Combined Joint Dislocation; OF, Open Fracture: FT, Fracture Type; PHb, Postoperative Hemoglobin; OT, Operation Time; SSI, Surgical Site Infection; PPLAFE, Postoperative Pain Level After Functional Exercise; PFEC, Postoperative Functional Exercise Compliance; TAI, Traffic accident injury, UAF, Unilateral ankle fracture; BAF, Bilateral ankle fracture; TAF, Trimalleolar ankle fracture.

**Figure 8:**
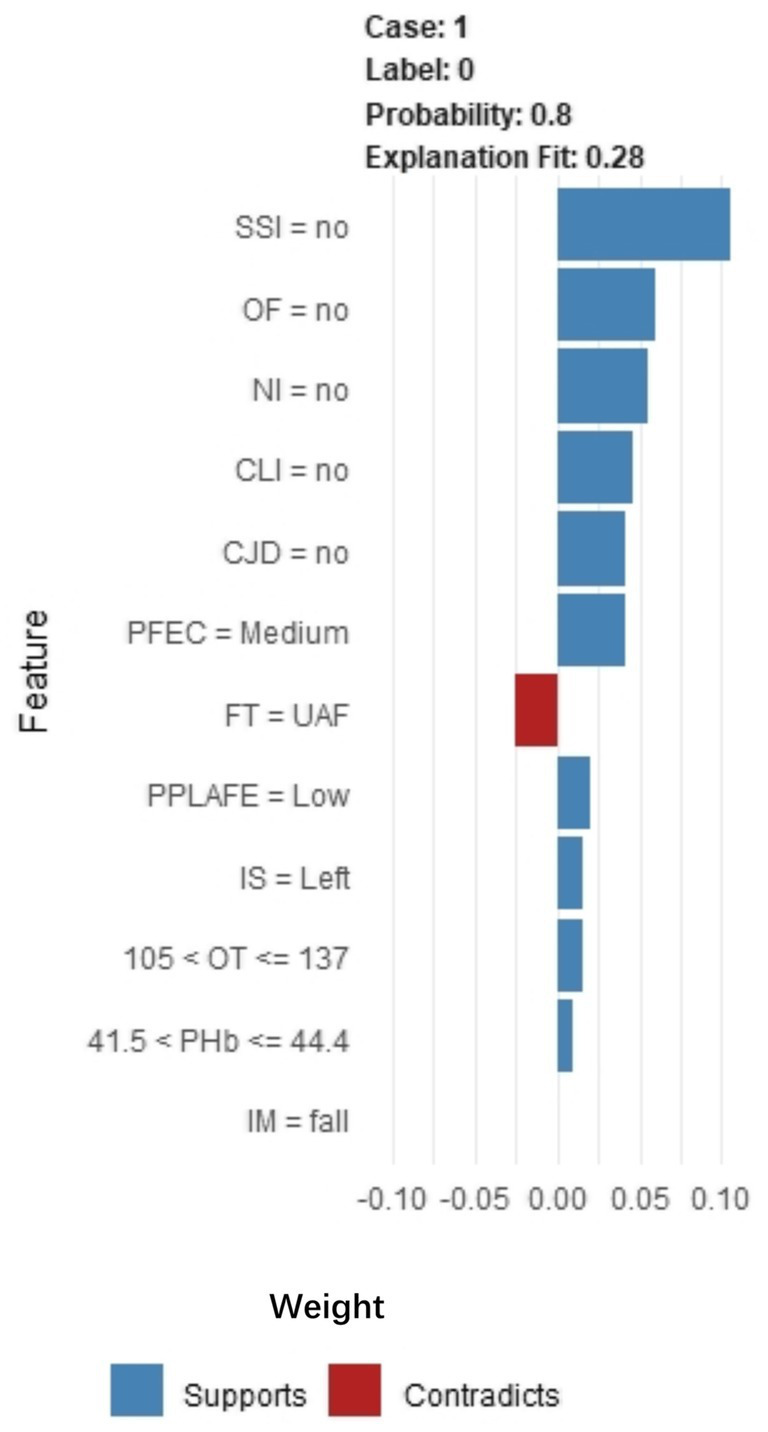
LIME algorithm explains individual prediction results plot. Parsed as an example of an ankle fracture. The picture shows the predicted expected probability of 80% poor postoperative recovery, estimated by the Lasso-Stacking model. This probability was determined by the predictive model. The length of each feature bar is proportional to the weight of that feature in the prediction. Longer bars represent features that contribute more to the predicted outcome. IM, Injury Mechanism; IS, Injury Site; CLI, Combined Ligament Injury; NI, Nerve Injury; CJD, Combined Joint Dislocation; OF, Open Fracture; FT, Fracture Type; PHb, Postoperative Hemoglobin; OT, Operation Time; SSI, Surgical Site Infection; PPLAFE, Postoperative Pain Level After Functional Exercise; PFEC, Postoperative Functional Exercise Compliance; TAI, Traffic accident injury; UAF, Unilateral ankle fracture; BAF, Bilateral ankle fracture; TAF, Trimalleolar ankle fracture.

Through SHAP analysis, clinical practitioners can gain insights into the decision-making basis of the lasso-stacking model. Figure 7A presents a SHAP summary plot where feature variables are sorted based on SHAP importance, from highest to lowest. Figure 7B is a bar chart where feature variables are arranged according to the mean absolute SHAP value, also in descending order. The SHAP analysis reveals that the variables, in terms of importance from greatest to least, are as follows: functional exercise compliance, concomitant ligament injury, open fracture, nerve injury, fracture severity, injury mechanism, concomitant joint dislocation, postoperative albumin level, pain level after postoperative rehabilitation exercise, operation duration, injury site, and postoperative infection. Figure 7C illustrates the SHAP plots for categorical variables, while Figure 7D showcases the SHAP plots for continuous variables. Figure 7E demonstrates the contribution of various features to the model’s prediction for a single sample of an ankle fracture patient who did not experience poor recovery after surgery. Meanwhile, Figure 8 utilizes the LIME algorithm to provide additional explanations for individual prediction results, complementing the interpretability analysis offered by SHAP.

## 4 Discussion

In this study, the ankle function of patients 3 months after ankle fracture surgery was evaluated using the AOFAS scale. It was found that 33.1% of patients scored less than 75 on the AOFAS, indicating poor joint function recovery after surgery. Despite continuous improvements in surgical techniques and rehabilitation exercises for ankle fractures in recent years, the incidence of poor joint recovery after surgery remains high. Therefore, early identification of high-risk groups and prompt implementation of relevant intervention measures for these groups are key to promoting good joint function recovery.

In this study, we constructed risk prediction models for poor functional recovery after ankle fracture surgery using five common machine learning algorithms: random forest, XGBoost, SVM, logistic regression, and lasso-stacking. After evaluating each model using metrics such as AUC, we found that all models were capable of predicting the occurrence of poor joint recovery after ankle fracture surgery to some extent, but there were significant differences in their predictive performance. The choice of machine learning algorithm mainly depends on the distribution of feature variables and model fitness (41). Logistic regression, as the simplest and most basic machine learning algorithm, performs well in predicting linear relationships between its characteristic variables and outcomes but is less effective for predicting non-linear relationships. In this study, it demonstrated the worst predictive performance. Random forest, a type of bagging integration algorithm, combines multiple decision trees. It constructs a random forest by integrating multiple decision trees and makes predictions based on the voting results of the random forest (42). In this study, the performance of the random forest is relatively stable. Compared to other models, the random forest model excels particularly when analyzing higher-dimensional data (43), which was well demonstrated in this study. Its overall predictive performance ranked second only to the lasso-stacking model. XGBoost may have advantages in predicting higher-dimensional data, but it may perform poorly in lower-dimensional datasets, with only 12 feature variables in this case (40). This limitation likely prevented it from fully leveraging its algorithmic strengths in this study. SVM operates by solving a hyperplane that can correctly classify the training data and maximize the geometric margin, making it effective in handling binary classification problems (44). This is well reflected in our study, where its overall predictive performance is slightly inferior to the lasso-stacking and random forest algorithms. Finally, the lasso-stacking model exhibited the best predictive performance in this study. The stacking model used in this study integrates the four different types of base models mentioned above, resulting in improved performance of the fused model. Under the fusion framework, the previously output results are input into a second-layer learner to obtain a better-performing prediction model. The lasso-stacking model in this study effectively learns from the advantages of the first four models and performs better in predictive performance than the first four models. Therefore, it is more suitable for predicting the recovery of patients’ joints after ankle fracture surgery. Current evaluation of post-ankle fracture joint function predominantly relies on the American Orthopaedic Foot & Ankle Society (AOFAS) score. While this instrument effectively assesses postoperative functional recovery, it lacks prospective predictive capability. To address this limitation, our study developed and compared five machine learning models, ultimately selecting the lasso-stacking ensemble as the optimal solution. This model leverages preoperative and perioperative clinical indicators from hospitalized patients to predict 3-month postoperative functional outcomes, providing actionable insights for clinicians to tailor individualized rehabilitation protocols. The lasso-stacking algorithm demonstrated superior discriminative performance, achieving area under the ROC curve (AUC) values of 0.877 (training set) and 0.791 (test set). Model robustness was rigorously validated through stratified analyses incorporating random data partitioning, demographic stratification, and fracture-type subgrouping, confirming consistent predictive stability across heterogeneous patient cohorts.

Through interpretive analysis of the optimal model, this study reveals that postoperative functional exercise compliance is the most critical factor influencing joint function recovery for patients after ankle fracture surgery. Numerous studies have shown (45–47) that early rehabilitation exercise is essential for the recovery of joint function after ankle fracture surgery. However, most clinicians currently focus on improving rehabilitation methods while paying little attention to patient compliance with these exercises. This has led to poor joint function recovery in some patients despite receiving feasible rehabilitation training guidance. Therefore, in clinical practice, besides instructing patients on correct rehabilitation methods, it is also crucial to urge them to perform effective exercises, thus reducing the risk of poor postoperative joint function recovery.

Additionally, fracture-related factors such as associated ligament damage, nerve damage, joint dislocation, injury mechanism, open fractures, injury location, and fracture severity also significantly impact joint function recovery after ankle fracture surgery. Among these, associated ligament damage is the most critical, possibly due to the instability it causes in joint function and the increased risk of ligament rerupture with early and extensive functional exercises (11, 48). This can lead to a fear of postoperative exercises among some patients, affecting their recovery. Hence, such patients require closer follow-up and individualized exercise plans.

Furthermore, postoperative albumin levels and operation time can also affect joint function recovery. According to SHAP, low postoperative albumin levels and prolonged surgical time increase the risk of poor joint function recovery. Patients with low postoperative albumin often have poorer nutritional status and may experience weakness, affecting their rehabilitation efforts (49, 50). In addition, albumin also plays roles in anti-oxidation, inflammation regulation, and immune response modulation (51, 52). In the state of hypoalbuminemia, the human body is more prone to trigger systemic inflammatory responses and immune suppression,resulting in elevated levels of C-reactive protein (CRP) and interleukin-6 (IL-6) in the body. This can cause tissue edema and stiffness around the joint, increase the risk of incision or joint cavity infections, and delay the recovery process. In addition, albumin plays a key role in maintaining plasma colloid osmotic pressure (52). A reduction in albumin levels can lead to interstitial fluid retention, resulting in postoperative limb swelling, stiffness, and pain, which in turn may inhibit patients’ willingness to perform functional exercises. Albumin deficiency can also affect collagen synthesis, cell proliferation, and matrix reconstruction (53), which will lead to the decline of the regeneration ability of fibroblasts, muscle cells, and chondrocytes, thus affecting wound healing and tissue repair and directly hindering the recovery of joint function. Therefore, it is an important factor that should receive more attention from clinical workers. Longer surgical procedures can lead to increased intraoperative bleeding and a higher risk of postoperative malnutrition. In addition, long-term surgery leads to soft tissue exposure, stretching, and electric coagulation for a long time, which aggravates local inflammation and scar formation, and affects the functional recovery of muscles and ligaments. Furthermore, prolonged stretch or compression can lead to muscle ischemia–reperfusion injury and impair local tissue activity. Prolonged use of anesthetics (especially neuromuscular blocking agents) may affect postoperative nerve activation and muscle tone recovery and delay the progress of functional exercise. Finally, prolonged operation time may increase the risk of infection and complications. Excessive pain during postoperative rehabilitation exercises can also cause fear among patients, disrupting their rehabilitation plans and affecting joint recovery (12, 54).

Finally, while it is widely known that postoperative infection can impact recovery and limb function, its influence was found to be relatively minor in this study. This may be due to advancing surgical techniques in recent years, which have reduced the incidence of postoperative infections and subsequent poor recovery, thereby diminishing the significance of this factor.

In this study, the development of a risk prediction model for postoperative poor joint function recovery in patients with ankle fractures is innovative. The constructed model demonstrated strong performance and can be preliminarily applied to support early and accurate prevention and control in clinical practice. In addition, interpretability analysis of the model identified important factors influencing poor joint function recovery in patients with ankle fracture after surgery, which provides some insights for clinical workers to intervene early. Finally, this study may serve as a foundation for future research on causal relationships and intervention strategies. However, there are certain limitations in this study. First, as a single-center study, it only validated internal data without external validation of the model, which may have an impact on the generalizability and robustness of the findings. Second, some data were incomplete, such as the degree of limb swelling and rehabilitation exercise methods, which were not included in the analysis. Previous studies have shown (15, 18) that rehabilitation exercises involving removable ankle support and different exercise duration can influence the recovery of joint function after ankle fracture surgery. However, this type of data is not optimized at present, and the lack of this metric may have an impact on the performance of the model. Finally, this study did not consider the changes in patients’ characteristics after discharge. Therefore, in the future, it is necessary to strengthen regional cooperation, further optimizing data collection and processing, continuously refining the model for different population data, and improving its accuracy. After multi-dimensional verification of the model, a visual early warning platform can be constructed to enable clinical workers to quickly identify high-risk patients and implement accurate prevention and control in the early stage.

## 5 Conclusion

Finally, this study developed five machine learning models and found that the lasso-stacking model showed the best performance, making it the most suitable for predicting high-risk populations with poor joint function recovery after ankle fracture surgery. Explanatory analysis of this model helped clarify its decision-making basis. In the future, the model should be verified and improved through multi-center external validation to support its application in clinical practice.

## Data Availability

The original contributions presented in the study are included in the article/Supplementary material; further inquiries can be directed to the corresponding authors.
