# Supplementary material for: A risk prediction model for poor joint function recovery after ankle fracture surgery based on interpretable machine learning
Source: Front Med (Lausanne). 2025 Jun 26;12:1553274. doi: 10.3389/fmed.2025.1553274 (PMC12241071; doi:10.3389/fmed.2025.1553274)
Supplement: Supplementary file 1 [file Data_Sheet_1.pdf]

Table S1. Performance of five machine learning-based models for predicting poor joint recovery after ankle fracture in the data set 1

Model	AUC（95%CI）	Accuracy	Kappa	Precision	Specificity	Sensitivity	F1 score
**RF**	0.797（0.738-0.856）	0.726	0.417	0.570	0.735	0.707	0.631
**LR**	0.596（0.514-0.678）	0.597	0.183	0.426	0.589	0.613	0.503
**SVM**	0.799（0.735~0.863）	0.743	0.493	0.573	0.669	0.893	0.698
**XGBoost**	0.649（0.573~0.725）	0.655	0.275	0.485	0.669	0.627	0.547
**Lasso-Stacking**	0.833（0.779~0.886）	0.755	0.464	0.622	0.795	0.68	0.65

Abbreviations:AUC, area under the curve;95%CI, confidence interval

Table S2. Performance of five machine learning-based models for predicting poor joint recovery after ankle fracture in the data set 2

Model	AUC（95%CI）	Accuracy	Kappa	Precision	Specificity	Sensitivity	F1 score
**RF**	0.849(0.814-0.884)	0.781	0.532	0.636	0.778	0.786	0.703
**LR**	0.714（0.668~0.760）	0.676	0.333	0.506	0.661	0.705	0.589
**SVM**	0.840（0.802-0.878）	0.744	0.494	0.572	0.670	0.896	0.689
**XGBoost**	0.732（0.686~0.778）	0.697	0.363	0.531	0.698	0.694	0.602
**Lasso-Stacking**	0.867（0.833~0.901）	0.824	0.613	0.712	0.843	0.786	0.747

Abbreviations:AUC, area under the curve;95%CI, confidence interval

Table S3. AUC Delong test results of five machine learning methods in training set, test set, data set 1 and data set 2

Model	Chronologically Split Data Set		Randomly Split Data Set		*z*	*P*
	AUC	95%CI	AUC	95%CI		
**RF**	0.840①	0.807~0.874①	0.797③	0.738-0.856③	1.467	0.143
			0.849④	0.814-0.884④	-0.049	0.961
	0.779②	0.698~0.860②	0.797③	0.738-0.856③	-0.360	0.719
			0.849 ④	0.814-0.884④	-1.555	0.121
**LR**	0.672①	0.626~0.719①	0.596③	0.514-0.678③	1.587	0.113
			0.714④	0.668~0.760④	-1.266	0.206
	0.691②	0.603~0.779②	0.596③	0.514-0.678③	1.558	0.120
			0.714④	0.668~0.760④	-0.460	0.646
**SVM**	0.809①	0.770~0.848①	0.799 ③	0.735~0.863③	1.041	0.299
			0.840④	0.802-0.878④	-1.054	0.292

	0.768②	0.686~0.851②	0.799③	0.735~0.863③	-0.070	0.944
			0.840④	0.802-0.878④	1.001	0.318
**XGBoost**	0.734①	0.692~0.776①	0.649③	0.573~0.725③	1.072	0.284
			0.732④	0.686~0.778④	0.095	0.924
	0.748②	0.663~0.832②	0.649③	0.573~0.725③	1.726	0.085
			0.732④	0.686~0.778④	0.325	0.745
**Lasso-Stacking**	0.877①	0.847~0.906①	0.833③	0.779~0.886③	1.251	0.212
			0.867④	0.833~0.901④	0.205	0.838
	0.791②	0.711~0.871②	0.833③	0.779~0.886③	-0.671	0.503
			0.867④	0.833~0.901④	-1.529	0.128

Abbreviations:AUC, area under the curve;95%CI, confidence interval;①,training set;②,testing set③,data set one; ④,data set two

Table S4. Performance of five machine learning-based models for predicting poor joint recovery after ankle fracture in different gender groups

Group	Model	AUC（95%CI）	Accuracy	Kappa	Precision	Specificity	Sensitivity	F1 score
	**RF**	0.784(0.7373-0.830)	0.742	0.414	0.631	0.824	0.582	0.605
	**LR**	0.65(0.592~0.708)	0.659	0.013	0.500	0.989	0.021	0.041
**Man**	**SVM**	0.565(0.505~0.625)	0.374	-0.005	0.339	0.114	0.879	0.489
	**XGBoost**	0.733(0.684~0.782)	0.703	0.346	0.561	0.762	0.589	0.574
	**Lasso-Stacking**	0.796(0.761~0.842)	0.659	0.354	0.500	0.577	0.858	0.632
	**RF**	0.772(0718~0.826)	0.753	0.438	0.607	0.808	0.636	0.621
	**LR**	0.650(0.589~0.710)	0.693	0.057	0.833	0.996	0.047	0.089
**Female**	**SVM**	0.525(0.455~0.594)	0.348	-0.032	0.307	0.122	0.832	0.488
	**XGBoost**	0.715(0.656~0.774)	0.708	0.331	0.541	0.782	0.551	0.546
	**Lasso-Stacking**	0.773(0.717~0.828)	0.696	0.389	0.515	0.651	0.794	0.625

Abbreviations:AUC, area under the curve;95%CI, confidence interval

Table S5. Performance of five machine learning-based models for predicting poor joint recovery after ankle fracture in different fracture type groups

Group	Model	AUC（95%CI）	Accuracy	Kappa	Precision	Specificity	Sensitivity	F1 score
	RF	0.827(0.793~0.860)	0.750	0.484	0.625	0.741	0.767	0.689
	LR	0.670(0.625-0.715)	0.633	0.277	0.494	0.584	0.721	0.586
UAF	SVM	0.817(0.781~0.854)	0.725	0.472	0.575	0.620	0.912	0.710
	XGBoost	0.720(0.677~0.762)	0.675	0.349	0.535	0.639	0.740	0.621
	Lasso-Stacking	0.853(0.821~0.885)	0.762	0.519	0.627	0.720	0.837	0.717
	RF	0.835(0.700~0.970)	0.605	0.293	0.440	0.462	0.917	0.595
	LR	0.647(0.435~0.860)	0.605	0.211	0.421	0.577	0.667	0.516
BAF	SVM	0.923(0.839~1.000)	0.842	0.678	0.667	0.769	1.000	0.800
	XGBoost	0.418(0.184-0.649)	0.526	-0.049	0.286	0.615	0.333	0.308
	Lasso-Stacking	0.952(0.889~1.000)	0.868	0.726	0.706	0.808	1.000	0.828
	RF	0.879(0.798~0.960)	0.887	0.599	0.722	0.947	0.619	0.667
	LR	0.608(0.472~0.744)	0.765	0.133	0.312	0.883	0.238	0.270
TAF	SVM	0.781(0.638~0.923)	0.809	0.442	0.483	0.840	0.667	0.560
	XGBoost	0.612(0.483-0.741)	0.783	0.126	0.333	0.915	0.190	0.242
	Lasso-Stacking	0.894(0.826-0.963)	0.870	0.586	0.625	0.904	0.714	0.667

Abbreviations:AUC, area under the curve;95%CI, confidence interval; Unilateral ankle fracture: UAF; Bilateral ankle fracture: BAF; Trimalleolar ankle fracture: TAF

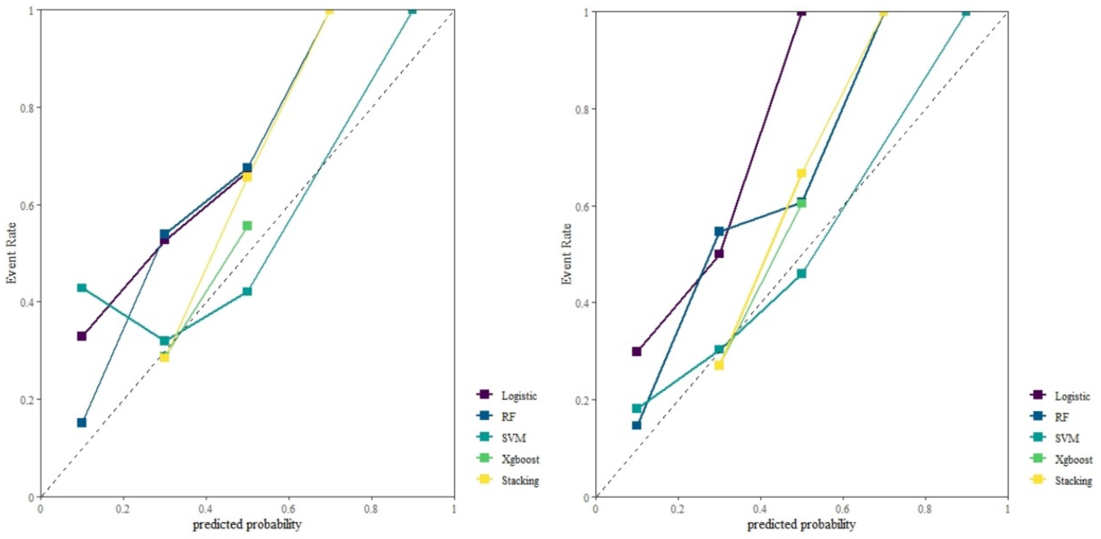

A B

Fig S1. Model calibration curve；(A): model calibration curves in man；(B) Model calibration curves in female

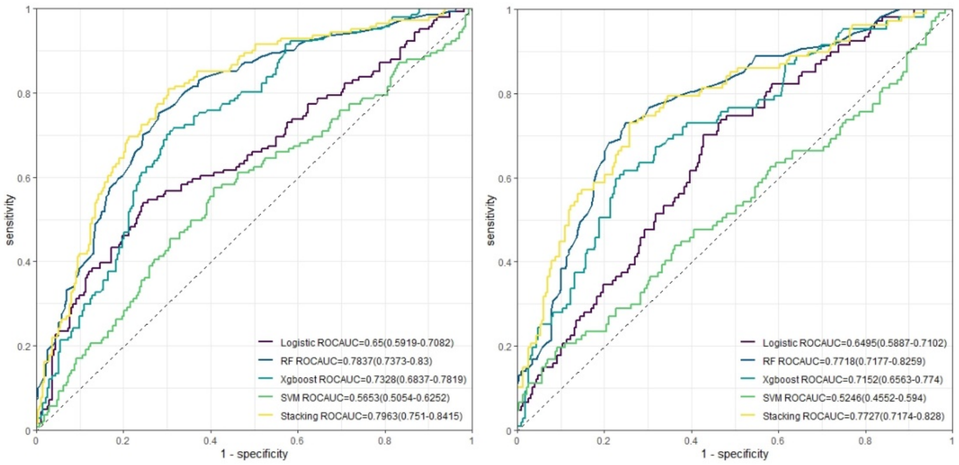

A B

Fig S2. Model ROC curve; (A): ROC curve of the model in man;(B): ROC curve of the model in female; ROC: receiver operating characteristic

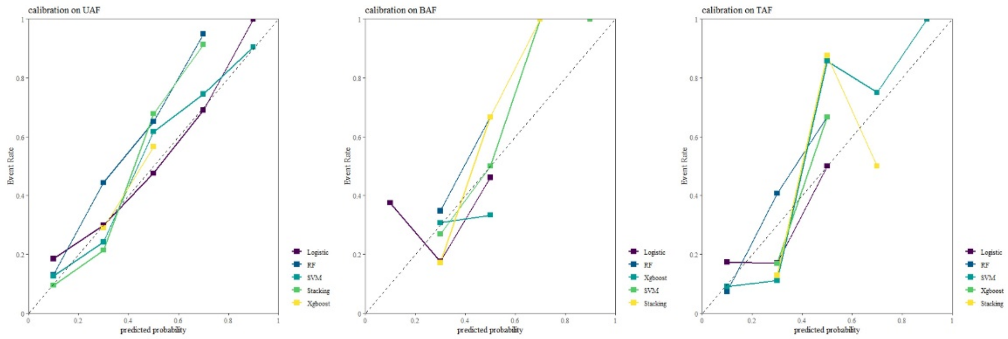

A B C

Fig S3. Model calibration curve；(A): model calibration curves in Unilateral ankle fracture；(B) Model calibration curves in Bilateral ankle fracture; (C) Model calibration curves in Trimalleolar ankle fracture; Abbreviations: UAF: Unilateral ankle fracture ;BAF: Bilateral ankle fracture TAF: Trimalleolar ankle fracture.

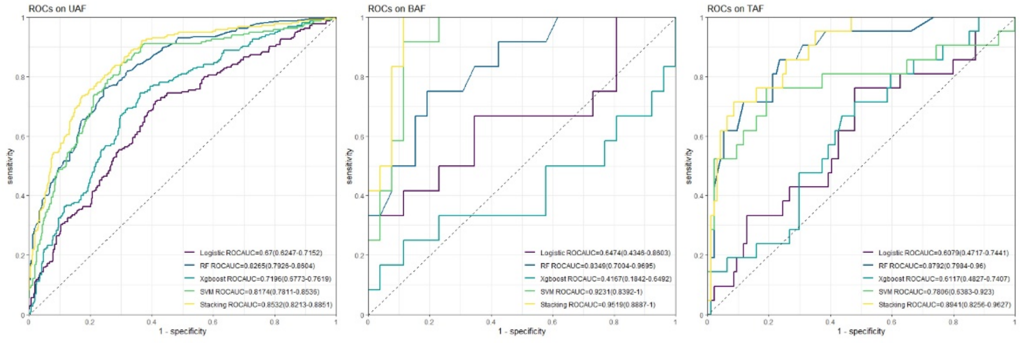

A B C

Fig S2. Model ROC curve; (A): ROC curve of the model in Unilateral ankle fracture;(B): ROC curve of the model in Bilateral ankle fracture; (C): ROC curve of the model in Trimalleolar ankle fracture; Abbreviations: UAF: Unilateral ankle fracture ;BAF: Bilateral ankle fracture TAF: Trimalleolar ankle fracture; ROC: receiver operating characteristic.
